# Are hygiene and public health interventions likely to improve outcomes for Australian Aboriginal children living in remote communities? A systematic review of the literature

**DOI:** 10.1186/1471-2458-8-153

**Published:** 2008-05-08

**Authors:** Elizabeth McDonald, Ross Bailie, David Brewster, Peter Morris

**Affiliations:** 1 Menzies School of Health Research, Institute of Advanced Studies, Charles Darwin University, Darwin, Northern Territory, Australia; 2 James Cook University, Cairns, Queensland, Australia; 3 Northern Territory Clinical School, Flinders University, Adelaide, South Australia, Australia

## Abstract

**Background:**

Australian Aboriginal children living in remote communities still experience a high burden of common infectious diseases which are generally attributed to poor hygiene and unsanitary living conditions. The objective of this systematic literature review was to examine the epidemiological evidence for a relationship between various hygiene and public health intervention strategies, separately or in combination, and the occurrence of common preventable childhood infectious diseases. The purpose was to determine what intervention/s might most effectively reduce the incidence of skin, diarrhoeal and infectious diseases experienced by children living in remote Indigenous communities.

**Methods:**

Studies were identified through systematically searching electronic databases and hand searching. Study types were restricted to those included in Cochrane Collaboration Effective Practice and Organisation of Care Review Group (EPOC) guidelines and reviewers assessed the quality of studies and extracted data using the same guidelines. The types of participants eligible were Indigenous populations and populations of developing countries. The types of intervention eligible for inclusion were restricted to those likely to prevent conditions caused by poor personal hygiene and poor living environments.

**Results:**

The evidence showed that there is clear and strong evidence of effect of education and handwashing with soap in preventing diarrhoeal disease among children (consistent effect in four studies). In the largest well-designed study, children living in households that received plain soap and encouragement to wash their hands had a 53% lower incidence of diarrhoea (95% CI, 0.35, 0.59). There is some evidence of an effect of education and other hygiene behaviour change interventions (six studies), as well as the provision of water supply, sanitation and hygiene education (two studies) on reducing rates of diarrhoeal disease. The size of these effects is small and the quality of the studies generally poor.

**Conclusion:**

Research which measures the effectiveness of hygiene interventions is complex and difficult to implement. Multifaceted interventions (which target handwashing with soap and include water, sanitation and hygiene promotion) are likely to provide the greatest opportunity to improve child health outcomes in remote Indigenous communities.

## Background

In the Northern Territory (NT) an Australian Indigenous infant aged between four weeks and one year is seven times more likely to be admitted to hospital than a non-Indigenous child of the same age. The majority of these admissions are for respiratory, diarrhoeal and parasitic diseases (69%), while the average number of conditions associated with each episode of hospitalisation is 2.7 [1]. This high burden of preventable respiratory, enteric, ear, eye and skin infectious disease is largely attributed to unsatisfactory living conditions and poor personal hygiene. Household crowding leads to more frequent interpersonal contact and increases the risk of cross infection [2]. High burdens of infection, combined with inadequate nutrition, are considered to account for approximately 50% of all cases of anaemia among NT Indigenous children aged less than five years. Growth data reveal wasting rates of 4–8% and stunting rates of 15–17% with these rates comparable to those of children of the same age living in Thailand [1]. Chronic Suppurative Otitis Media (CSOM) is very common [3] and Bronchiectasis is not uncommon [4]. In children under three years of age repeated acute episodes or chronic states of otitis media interfere with normal speech and language development, while some permanent hearing loss often results [5]. Scabies and Group A Streptococcal pyoderma is endemic among Indigenous Australian children living in remote communities [6-8] and as a result the prevalence of post-Streptococcal glomerulonephritis is high [9]. This high level of exposure to Group A Streptococci is also responsible for the continuing high rate of rheumatic fever and rheumatic heart disease among these children [10]. It is claimed that trachoma rates for Indigenous Australian children in some remote communities have not changed over the past 20 years [11]. Australia is now the only developed country in the world that has not eradicated this disease.

There has been little research into the problem of poor hygiene and unsanitary living conditions in remote Australian Aboriginal communities and the benefits of population-level interventions. Efforts to improve Aboriginal children's health have to date mostly focused on the treatment or eradication of diseases by the use of vaccines and improved medical case management. The impact of poor personal, domestic and community hygiene on children's health has largely been ignored. Taking this approach could have contributed to the slow rate of improvement in Aboriginal child health. The primary objective of this systematic literature review was to examine the epidemiological evidence of effectiveness of hygiene and public health intervention strategies, separately or in combination, on the occurrence of common preventable childhood infectious diseases. The purpose of the review was to inform the development of hygiene improvement programs which aim to reduce the incidence of skin, diarrhoeal and respiratory diseases (including otitis media) experienced by children living in remote Indigenous communities in central and northern Australia. Hence, the approach taken in this systematic review is more broadly based than reviews that focus on the efficacy of a single intervention.

The Australian Indigenous population represents only 1–2% of the total Australian population of approximately 20 million people [12]. In the NT approximately 28.5% (51,876) of the total population is indigenous and approximately 71% of this group live in geographical locations considered very remote, that is, geographic distance which imposes the highest restriction upon accessibility to the widest range of goods, services and opportunities for social interaction [13]. In the NT there are numerous small remote communities ranging in size from a single family group to 2500 people. Many of these communities are currently attempting to deal with serious health and social problems, for example the high prevalence of renal disease and diabetes and high levels of community and domestic violence due to drug and alcohol abuse [12,14,15].

Some distinctive demographic characteristics of NT Indigenous communities that impact on the health and welfare of children through socio-economic disadvantage include a dependency ratio of 66 children (0 – 14 years) for each 100 adults of working age (15 – 64 years) [1]. In the non-Indigenous population this ratio is 32:100. NT Indigenous females aged less than 15 years are 32 times more likely to give birth than NT non-Indigenous women of a similar age. There are a high number of one parent families (approximately 25%) [1]. Indigenous families in remote NT communities must survive on very low incomes in remote locations where the cost of the basic items necessary for daily life, for example food, personal soap, laundry detergent, cleaning products and tools and clothing, are very high [14,16].

Indigenous people living in the NT experience extreme disadvantage. The key underlying causes for this disadvantage are considered to be social inequality and powerlessness with these factors impacting negatively on their health and well being [14,16]. The declaration of *terra nullius *by the colonisers of Australia in 1788 led to the resident Indigenous population being viewed as savages to be dominated and eliminated [17]. This and subsequent government policies are regarded as important determinants of the present profile of Indigenous health [14]. These pressures have contributed to alcohol abuse, violence and poor physical and mental ill-health. Rapid social and subsequent cultural change has contributed to individual, family and community dysfunction among Australian Indigenous people [15].

Modern Australian history shows that since white contact Aboriginal culture has been widely denigrated by the Australian non-Indigenous population, in particular by government officials and administrators who had control over Indigenous affairs [18,19]. Indigenous people, when they are continually requested to change behaviours for health benefits, might perceive that cultural denigration is still occurring. Indigenous people have been distressed in the past by the use of 'victim blaming' theories to explain health program failures and are now wary of non-Indigenous persons implementing programs in sensitive domains. Engaging with communities to address preventative public health activities such as poor hygiene issues is difficult [20]. In the 1970s researchers forewarned of some of the public health risks that may eventuate if some child care practices related to a hunter-gatherer lifestyle continued unchanged in permanent settlements [21,22]. The child care practices that continue today that are seen to contribute to the failure of the health and hygiene needs of young children being met include: shared mothering; encouraging young children to be independent and to explore and come to terms with their environment; the expectation that mothers will not cause their children distress; and approaches to child care that largely focus on protecting children from the physical dangers in the environment. More recent research indicates that four child care practices in particular are important barriers to reducing rates of infection among children, including 1) the tradition of sharing responsibility for the day-to-day care of young children, and the degree of freedom very young children have in determining their own care that enables them to reject any hygiene training attempted by their carers; 2) children, in particular girls, are expected to meet the hygiene needs of infants and toddlers. These children themselves go unsupervised in meeting their own hygiene needs; 3) the generally accepted practice of young children defaecating in the open and the general acceptance of children's faeces in the environment; 4) the apparent lack of awareness around the risks posed to young children by other children's faeces and nose and ear discharges [23].

Historically, both Federal and Territory Governments have had responsibilities for directly delivering services such as housing related infrastructure, essential and municipal services and municipal infrastructure to remote Indigenous communities in the NT [24]. Complex housing programs and funding arrangements have lead to confusion at the community level. Disputes between Governments or between departments or divisions within a government over housing issues are not uncommon. Indigenous community councils struggle to maintain their existing housing stock in a satisfactory condition [25]. The high repair and maintenance needs of houses on remote Indigenous communities is attributed to overcrowding, the manner in which people live in houses, poor design, sub-standard construction, use of inappropriate technology, and the lack of finances and other resources to rectify problems [26]. Clark [27] states that environmental health officers in the NT clearly identified overcrowding, inadequate housing and health hardware as the major factors compromising personal and domestic hygiene in the remote Indigenous communities they visit. One environmental health officer reported that many of his colleagues felt unable to address behavioural issues around poor hygiene in remote communities until the physical environment in which people live enables healthy living practices. Some officers believe that housing can have an immediate impact and improve health.

Whereas others consider that even with functioning hardware, the current social and environmental conditions in many remote communities means that only the most extremely motivated would be able to sustain safe hygiene practises on a continual basis [28]. While the Northern Territory Health Service has always professed the importance of an educational approach as a strategy for addressing environmental health issues, Clark has observed that each attempt has been abandoned in the face of changing policies, conflicting paradigms of service delivery and economic restraint [27]. There is a general consensus that preventing infection in the household and community is a health priority for Australian Indigenous children living in remote communities [2-4,9,10]. However, despite the rhetoric around the need to protect the health of these children it does not often translate into meaningful action [29].

## Methods

### Data Sources

Using a key word search strategy studies were identified through searching electronic databases, including Medline, EMBASE, CINAHL, DARE, Cochrane Library and SCI up to 31 December 2003. A key word search of the World Wide Web (WWW) was completed. Journals were also hand searched and reference lists of included papers were scanned. The following key word strategy was used using the OVID interface: ((Hygiene OR (hygiene AND intervent$) OR (intervent$ AND stud$) OR prevent$ OR (primary AND prevent$) OR (public AND health) OR (health AND promotion) OR handwash$ OR (face AND wash$) OR (skin AND care) OR soaps.tw OR sanitation) NOT condoms NOT bednets NOT vitamin A AND (indigenous OR aborigin$ OR (ethnic AND group$) OR (developing AND countr$)).

### Inclusion Criteria

Study types were restricted to Randomised Controlled Trials (RCT) (including cluster randomisation); Clinical Controlled Trials (CCT); Controlled Before and After Studies (CBA); and Interrupted Time Series Analyses (ITS). The types of participants eligible were Indigenous populations and populations of developing countries not selected by specific risk factors or the presence of specific illness. The types of intervention eligible were education/health promotion; the introduction of hygiene hardware (for example, the building of latrines); housing infrastructure (for example, design features); the introduction of new behaviours or use of methods to change behaviour (for example, local government by-laws); and the introduction of hygiene aids (for example, personal soap). Interventions not eligible for inclusion in the review included studies of vaccine efficacy, drug therapies including Vitamin A, and use of bed nets and condoms. The outcome measures assessed were rates of diarrhoeal, skin, respiratory diseases and all illness; child growth parameters; and the degree/level of adoption of promoted behaviours. Outcomes were categorised according to the length of time the outcome/s were measured after initiation of the intervention. Published and unpublished studies in the English language were eligible for inclusion.

### The Review Process

The review process included two reviewers independently scanning the initial search results by title. Abstracts were then retrieved and divided into two groups with two reviewers independently scanning each abstract for studies considered to meet the review's eligibility criteria. Copies of the articles were obtained and four reviewers independently assessed each study. Discrepancies were resolved by discussion. Reviewers assessed the quality of studies and extracted data using guidelines and data extraction tools adapted from the Cochrane Collaboration Effective Practice and Organisation of Care Review Group (EPOC) [30]. Interventions were categorised by type and the review data were analysed for each intervention category.

## Results

**Figure 1:**
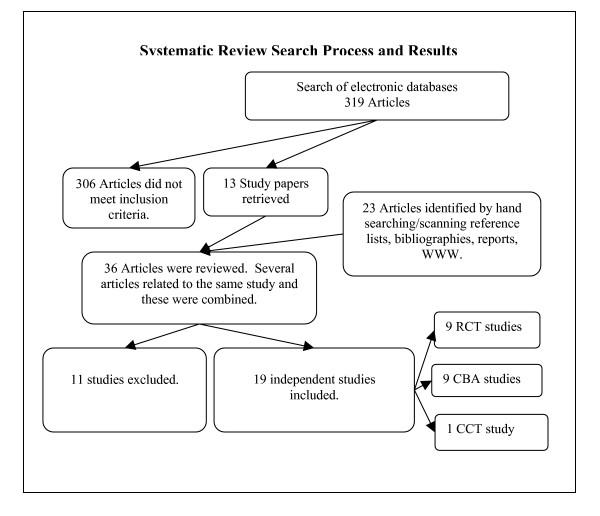
Overview of search process and search results.

Altogether 342 potentially relevant papers were identified. Based on the information provided in their abstracts, 306 papers were considered not to meet the review's eligibility criteria. Thirty-six papers were reviewed in full. Only 19 studies were considered by reviewers to meet the review's eligibility and quality inclusion criteria (Figure 1).

Table 1 lists the 19 studies included in the review. In some cases it was necessary to extract information from more than one published report to obtain all the information necessary to appraise studies.

**Table 1:** List of included studies.

No	Study	Study Design
1	Domestic transmission routes of pathogens: the problem of in-house contamination of drinking water during storage in developing countries [31]	CCT^a^
2a	An educational intervention for altering water-sanitation behaviours to reduce childhood diarrhoea in urban Bangladesh: formulation, preparation and delivery of educational intervention [32]	RCT^b^
2b	An educational intervention for altering water-sanitation behaviours to reduce childhood diarrhoea in urban Bangladesh. II. A randomized trial to assess the impact of the intervention on hygienic behaviours and rates of diarrhoea [33]	
3	Chlorination and safe storage of household drinking water in developing countries to reduce waterborne disease [34]	RCT
4	An intervention for the promotion of hygienic faeces disposal behaviours in a shanty town of Lima, Peru [35]	RCT
5a	Handwashing intervention to reduce ascariasis in children [36]	RCT
5b	Prevention of diarrhoea and dysentery by handwashing [37]	
6a	Use of insecticide for fly control reduced the incidence of childhood diarrhoea in rural Pakistan [38]	RCT
6b	Impact of fly control on childhood diarrhoea in Pakistan: community-randomised trial [39]	
7a	Sustainability of a water, sanitation and hygiene education project in rural Bangladesh: a 5-year follow-up [40]	RCT
7b	Lack of impact of a water and sanitation intervention on the nutritional status of children rural Bangladesh [41]	
7c	Reduction in diarrhoeal diseases in children in rural Bangladesh by environmental and behavioural modifications [42]	
8	Hand washing with soap reduces diarrhoea and spread of bacterial pathogens in a Bangladesh village [43]	CBA^c^
9	Effect of fly control on trachoma and diarrhoea [44]	CBA
10	Impact of face-washing on trachoma in Kongwa Tanzania [45]	RCT
11	Diarrhoea prevention through household-level water disinfection and safe storage in Zambia [46]	CBA
12	Keeping clean water clean in a Malawi refugee camp: a randomized intervention trial [47]	RCT
13	A longitudinal study of the impact of behavioural change intervention on cleanliness, diarrhoeal morbidity and growth of children in rural Bangladesh [48]	CBA
14	Measuring the effect of a hygiene behaviour intervention by indicators of behaviour and diarrhoeal disease [49]	CBA
15	Effect of intensive handwashing promotion on childhood diarrhoea in high-risk communities in Pakistan: a randomized controlled trial [50]	RCT
16	Hand-washing reduces diarrhoea episodes: A study in Lombok, Indonesia [51]	CBA
17a	The Imo State (Nigeria) Drinking Water Supply and Sanitation Project, 2. Impact on dracunculiasis, diarrhoea and nutritional status [52]	CBA
17b	The Imo State (Nigeria) Drinking Water Supply and Sanitation Project, 1. Description of the project, evaluation methods, and impact on intervening variables [53]	
18	Community-based hygiene education to reduce diarrhoeal disease in rural Zaire: impact of the intervention on diarrhoeal morbidity [54]	RCT
19	A Longitudinal Study of the Impact of Village Health Education on Environmental Sanitation [55]	CBA

^a^Clinical Control Trial; ^b^Randomised Control Trial; ^c^Controlled Before and After.

Eleven studies were considered not eligible for inclusion and the reasons for this include: three were cross sectional studies [56-58]; one an ethnographic study [59]; two studies did not include the use of a control group [60,61]; two papers were reviews [62,63]; one paper only provided information on study design and no results [64]; one report did not include baseline data [65]; and one study (a RCT in which the participants were Australian Indigenous children) included eye washing as a means to treat children with a clinical diagnosis of trachoma [66] and was thus therapeutic rather than preventive.

### The Characteristics of Included Studies

No studies were identified in which the participants were Indigenous people living in developed countries. In all the studies the participants belonged to disadvantaged groups living in developing countries. The interventions were grouped according to six intervention categories (Table 2). Interventions involving education and hygiene behavioural change were further divided into two categories: 1) education focusing on promoting handwashing with soap; and 2) education to achieve other hygiene behavioural change. Interventions in the latter category include hygiene education programs that delivered two or more safe hygiene practice messages (4 studies), washing dishes immediately after eating meals (1 study), and promoting the use of potties by young children (1 study). The other categories include education and face washing (1 study), insecticide spraying for fly control (2 studies), water supply, sanitation and hygiene education (2 studies) and improved water storage in the home (4 studies). The study of the intervention involving education and face washing was kept separate as face washing occurred in conjunction with a mass treatment program for trachoma.

**Table 2:** Number of studies in each intervention group and those with diarrhoeal disease as an outcome.

Intervention	Nos of Studies	Nos Of Studies with Diarrhoeal Disease as an Outcome
Education and hand washing with soap	4	4
Education and other hygiene behaviour change	6	3
Education and face washing	1	-
Insecticide spraying for fly control	2	2
Water supply sanitation and hygiene education	2	2
Improved water storage in the home	4	3

The results of studies that reported decreased contamination of water stored in the home were not reviewed because 'water quality' is a large topic in itself and beyond the scope of this review. A Cochrane review on this topic has recently been published [67]. The search terms used captured only those studies with primary outcomes assessing health as opposed to outcomes measuring water quality *per se*.

Some studies include more than one primary outcome measure [34,36,37,40-42,44,51,55]. A primary outcome measure in 14 of the 19 nineteen reviewed studies was rate of diarrhoeal disease (Table 2). The interventions used with this primary outcome measure include education and handwashing with soap (4), education and other hygiene behaviour change (3), improving water storage in the home (3), insecticide spraying for fly control (2), water supply, sanitation and education (2).

Two studies [44,45] report rates of trachoma as their primary outcome measure and approached blocking the transmission of infection through two intervention strategies: 1) spraying of insecticide to control fly populations, and 2) education and face washing. Three studies [35,48,55] include behaviour change or knowledge attainment among their primary outcome measures, such as 1) knowledge and behaviour change around handwashing, 2) participant knowledge and behaviour change concerning the use of potties, and 3) knowledge and practice of a range of hygiene practices such as washing dishes after meals, animal control, the cleaning of latrines. In all but six studies [31,35,45,46,49,55] outcome measures are reported as they relate to the rates of disease among child participants. In some cases, data were collected on all members of households and results presented according to an age breakdown. There was inconsistency across the studies in relation to the age categories used in enrolling participants, in data analysis and in reporting of results.

All eligible studies originated from developing countries in tropical climates. Seasonal influence on rates of infection was relevant in all studies. However, the impact of seasonality on rates of infection was taken into account in only 11 of the 19 eligible studies.

Issues of effectiveness, appropriateness, affordability and cost effectiveness of interventions were investigated in all eligible studies. The authors of four papers [31,34,43,46] argue that the interventions used in their studies were not only effective, but the cost of the interventions were affordable by either poor families or by governments of developing countries. On the other hand, two studies [39,50] acknowledged that despite the proven effectiveness of their interventions, the approach used by them was not suitable for wider application due to high cost.

Assessment of sustained impact was based on effect lasting longer than one year (Level A) and equal to or less than one year (Level B). Only three of the 19 studies [40,45,54] reported measuring health and other indicators again after intervention activities had ceased. Time of follow-up after the intervention cessation ranged from six months to six years [40,45,54].

### Magnitude of Effect for Different Types of Intervention

In this section studies are grouped according to intervention type and results are provided in detail for health outcomes only. Quality scores for each study, assessed according to EPOC systematic review study design quality criteria, are provided along with study results. The seven quality indicators used to score RCT studies were randomisation, allocation concealment, comparable control, blinded assessment, baseline measurement, reliable primary outcome measure and protection against contamination. The six quality indicators used to score CBA studies were comparable control, blinded assessment, baseline measurement, reliable primary outcome measure, protection against contamination and follow-up of participants.

### Education and Handwashing with Soap

Four studies are included in this category: three [36,43,50] conducted in poor urban or peri-urban environments and one [51] in a rural village. The studies had some similarities, such as a small number of educational messages, diarrhoeal rates as a primary outcome, provision of soap at no cost to participants, and relatively intense home visiting. Obvious methodological differences in the studies include the manner in which data are categorised and analysed, the use of different process or intermediate outcomes, and different definitions for the same health outcome. The main results of studies listed under this category, along with the quality score for each study, are provided in Table 3.

**Table 3:** Study results – education and handwashing with soap

Study	Outcome	Relative Risk/Incident Density Ratio (95% CI)
Luby, Agboatwalla *et al *2004 [50]	*Diarrhoea*	
RCT	Mean Incidence	
Quality Score 6^a^	Antibiotic Soap	0.50 (0.36, 0.63)
	Plain Soap	0.47 (0.35, 0.59)
Shahid, Greenough *et al *1996 [43]	*Diarrhoea*	
CBA	Age groups	
Quality Score 1^b^	0 – 11 mths	0.39 (0.29, 0.54)
	12–23 mths	0.53 (0.37, 0.77)
	24–59 mths	0.44 (0.34, 0.59)
	6 – 9 yrs	0.27 (0.19, 0.37)
	10 – 14 yrs	0.28 (0.16, 0.49)
	Over 15 yrs	0.38 (0.30, 0.49)
	All	0.38 (0.33, 0.43)
Wilson, Chandler *et al *1991 [51]	*Diarrhoea*	
CBA	Children <11yrs	0.21 (0.08, 0.53)^c^
Quality Score 3^b^	*Skin/Eye Disease*	
	Children <11yrs	2.54^d^
Han, Hlaing *et al *1988, 1989 [36]	*Ascaris*	
RCT	Children 36–59mths	1.0^d^
Quality Score 4^a^	*Diarrhoea*	
	<2yrs	0.69 (0.48, 1.01)^e^
	≥ 2yrs	0.67 (0.45, 0.98)^e^
	All children <5yrs	0.70 (0.54, 0.92)^f^
	*Dysentery*	
	<2yrs	0.59 (0.22, 1.55)
	≥ 2yrs	1.21 (0.52, 2.80)
	All children <5yrs	0.93 (0.39, 2.23)

^a^Maximum score for RCT 7; ^b^Maximum Score for CBA 6; ^c^CI taken from Systematic Review data Fewtrell and Colford [71]; ^d^No confidence intervals provided; ^e^P < 0.05; ^f^P < 0.01.

### Education and Other Hygiene Behaviour Change

Six studies are included under this intervention category. Four studies were completed in a rural environment [48,49,54,55] and two took place in an urban context [32,33,35]. These studies all incorporate the use of educational strategies to try and effect changes in hygiene behaviour. One study utilised education and social marketing strategies [49]. The design of these studies included both qualitative and quantitative research methods. The information in Table 4 summarises the main results reported in these studies.

**Table 4:** Study Results – education and other hygiene behaviour change

Study	Outcome	Relative Risk (95% C I)
Pinfold and Horan 1996 [49] CBA Quality Score 3^a^	Fingertip contamination levels	

Haggerty, Muladi *et al *1994 [54] RCT Quality Score 4^b^	Risk of reporting diarrhoea in peak diarrhoeal season	0.89 (0.80, 0.98)^c^

Ahmed, Zeitlin *et al *1993 [48] CBA Quality Score 4^a^	*Diarrhoea*	
	Children 0 -18 months	0.66^d^
		
Stanton & Clemens 1987, 1987 [32,33] RCT Quality Score 4^b^	*Diarrhoea*	
	Total episodes Children <6yrs	0.74 (0.67, 0.82)^e^
	Age Group	
	0 yrs	0.76 (0.55, 1.05)
	1 yrs	0.92 (0.75, 1.13)
	2 yrs	0.54 (0.43, 0.66)^e^
	3 yrs	0.68 (0.54, 0.85)^f^
	4 yrs	0.93 (0.69, 1.25)
	5 yrs	0.92 (0.68, 1.21)
	Overall	0.75 (0.68, 0.83)^e;g^

Yeager, Huttly *et al *2002 [35] RCT Quality Score 3^b^	No disease outcome – Hygiene knowledge and practices	
		
Tonon 1982 [55] CBA Quality Score 2^a^	No disease outcome – Observed sanitary changes	

^a^Maximum Score for CBA 6; ^b^Maximum score for RCT 7; ^c^CI taken from Fewtrell and Colford [71]; ^d^Study not analysed as a CBA; RR taken from Fewtrell and Colford [71], no CI provided; ^e^P < 0.0001; ^f^P < 0.001; ^g^Episodes per 100 person-weeks of observation.

### Education and Face Washing

There was only one study in which face washing as an intervention followed a mass trachoma treatment program (Table 5). The outcomes measured included trachoma (severe vs. any) and the percentage of children with clean faces 12 months after the intervention.

**Table 5:** Study Results – education and face washing

Study	Outcome	Odds Ratio (95% C I)
West, Munoz *et al *1995 [45]	*Trachoma*	
RCT	Severe Trachoma	0.62 (0.40, 0.97)
Quality Score 4^a^	Any Trachoma	0.81 (0.42, 1.59)

^a^Maximum score for RCT 7.

### Insecticide Spraying to Control Flies

Two fly control studies took place in rural environments in different countries, and although the interventions were similar the study designs are quite different. In one study [38], the primary outcome was rates of diarrhoea among children, whereas the other [44] measured the effect of the intervention on trachoma and diarrhoeal rates among children (Table 6).

**Table 6:** Study Results – insecticide spraying to control flies.

Study	Outcome	Relative Risk (95% C I)
Chavasse, Shier *et al *1999 [38]	*Diarrhoea*	
RCT/Cross-over design	Mean rate (adjusted for 1 year)	0.77 (0.67, 0.89)^c^
Quality Score 5^a^		
Emerson, Lindsay *et al *1999 [64]	*Trachoma *(all ages)	
CBA	New active trachoma	0.25 (0.09, 0.64)^d^
Quality Score 3^b^	Active trachoma	0.39 (0.20, 0.77)^e^
	*Diarrhoea*	
	Children aged 3 – 60 months	
	Wet season	0.78 (0.64, 0.95)^f^
	Dry season	0.74 (0.34, 1.59)^g^

^a^Maximum score for RCT 7; ^b^Maximum Score for CBA 6; ^c^P = 0.007; ^d^P = < 0.003); ^e^P = 0.007; ^f^P = 0.01; ^g^P = 0.60.

### Water Supply, Sanitation and Hygiene Education

There are two such studies, both multi-interventional development projects involving the provision of hand-pumps, latrines and health education. One study [40-42] was completed in Bangladesh and the other in Nigeria [52,53]. For the Bangladeshi study different outcomes were reported in three separate publications [40-42]. For the Nigerian study information was obtained from two published papers [52,53]. The Nigerian study consisted of repeated cross-sectional surveys, which allows it to be treated as a CBA study. However, it is not clear that this study design was planned by the authors. The primary outcomes measured in the Bangladeshi study were rates of diarrhoea and child growth, whereas the Nigerian study measured rates of dracunculiasis and rates of diarrhoea. A summary of the results of both these studies is in Table 7.

**Table 7:** Study Results – water supply, sanitation and hygiene education

Study	Outcome	Relative Risk/Incident Density Ratio (95% C I)
1) Hoque, Juncker *et al *1996 [40] CBA Quality Score 2^a^	*Diarrhoea*	
	Children	
	<5 yrs	
	>5 yrs	*Point Prevalence provided^b^

2) Aziz, Hoque *et al *1990 [42] CBA	*Diarrhoea*	
	Children <5yrs	0.75 (0.70, 0.80)^c^

	*Dysentery*	
	Children <5yrs	0.73 (0.61, 0.88)^d^

3)Hasan, Briend *et al *1989 [41] CBA	Nutritional differences (no significant difference identified)	Not provided

1) Huttly, Blum *et al *1990 [52] 2) Blum, Emeh *et al *1990 [53] CBA Quality Score 2^a^	Diarrhoea – Children <6yrs	
	Village A/C	1.27^e^
	Village B/D	0.71^e^

^a^Maximum Score for CBA 6; ^b^<5yrs 1.96 (0.96, 2.78), >5yrs 2.25 (1.56, 3.23); ^c^P < 0.01; ^d ^P < 0.001; ^e^CI not provided.

### The Quality of the Evidence

Environmental and behavioural interventions are difficult to successfully implement and evaluate [68]. In this review not one study met every quality indicator of the EPOC guidelines. Assessment of effectiveness of interventions is complicated by the variable quality of studies [69] and lack of information on effectiveness of implementation. Only two studies reported problems with implementation and indicated that this had a negative impact on their outcomes [35,54]. None of the studies explicitly reported developing their intervention on published behaviour change or other theories. Seven studies were preceded by observational research activities in order to inform the intervention design and to refine their methodology [31,33,35,49,53-55].

Meta-analysis was not completed for studies in individual categories (or overall) due to the heterogeneity of interventions identified, and the generally poor quality of studies. Only one study [50] provided sound epidemiological evidence of effect. This RCT met six out of a possible seven study design quality indicators (randomisation, allocation concealment, comparable control, baseline measurement, reliable primary outcome measures and protection against contamination). The design of this study is likely to minimise its susceptibility to bias and provide the best estimate of effect. While it is reassuring that other studies describe similar effects the case for more high quality studies is overwhelming.

## Discussion

### Key Results

The underlying reason for conducting the review was to inform the development of hygiene improvement programs which aim to reduce the incidence of skin, diarrhoeal and respiratory diseases (including otitis media), conditions commonly experienced by children living in remote Indigenous communities in central and northern Australia. Of the nineteen studies considered eligible for inclusion in the review none included primary outcome measures associated with skin or respiratory diseases. Fourteen of the nineteen studies did include rates of diarrhoea as a primary outcome measure, reflecting the urgent need to reduce rates of child mortality from acute diarrhoeal disease in resource-poor countries.

There is clear and strong evidence of effect of education and handwashing with soap preventing diarrhoeal disease among children [50]. There is some evidence of effects of education and other hygiene behaviour change interventions, and of the provision of water supply, sanitation and hygiene education on reducing rates of diarrhoeal disease. The size of these effects is small and the quality of the studies generally poor. However, it may not be appropriate to disregard those interventions where the evidence of effect is small as there are a number of reasons why hygiene interventions may fail to show an impact on rates of infection. Included among these reasons are poor study design, implementation issues such as the short timeframe of most studies, and lack of flexibility of intervention implementation in the research design. In addition, some precondition may be required for an intervention to show some effect, for example, Esrey [58] found that providing an improved water supply was only beneficial to health when sanitation was also improved. Although the methodology used in this study has been questioned [70]. Studies of face washing and education and insecticide spraying to control flies to reduce rates of trachoma generally failed to show any significant evidence of effect. This is likely to be due to factors such as the endemic nature of the disease and the transmission occurring through multiple routes. Esrey's case in point is also likely to apply in this instance, for example, is it poor sanitation, the presence of live stock or another condition that creates the environment for flies to breed in large numbers?

**Figure 2:**
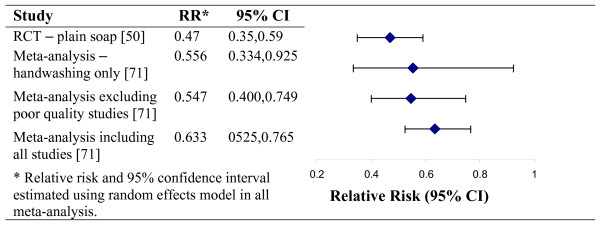
Comparison of key findings from recently published hygiene intervention systematic review and handwashing study.

The findings of this review are consistent with two recently published systematic reviews. One of these reviews investigated hygiene interventions to reduce diarrhoeal disease in less developed countries [71]. The authors included four additional studies which did not meet the criteria for our review; one was written in French, two were case-control studies, and the fourth study was a narrative account only. The high quality handwashing study by Luby et al [50] is not included in the Fewtrell and Colford [71] review presumably because it was published after their review was completed. The results of the meta-analysis and the later RCT are consistent (Figure 2).

The other systematic review assessed investigated face washing promotion as a means to reduce the prevalence of trachoma [72]. The authors of this review concluded that currently there is no evidence to support a beneficial effect of face washing alone or in combination with topical tetracycline in reducing active trachoma. Our findings are similar to this review and to those of a narrative review [73] on the effects of face washing and environmental changes to reduce the prevalence of trachoma which concluded that the promotion of face washing gave 'modest gains' for intense effort.

### Implications for Policy and Practice

This review aimed to cover issues of cost, intervention strategies, intervention integrity and ethical considerations and sustainability as well as size of the intervention effect with a view to maximising usefulness to policy makers and others working in the field. These issues are important to transfer research findings into policy and practice and for scaling up of interventions in different settings [74]. It is disappointing that the information available to do this to any meaningful extent is not provided in most study reports.

Since 1974, Governments in Australia have focused on providing housing and water and sanitation infrastructure to improve hygiene and living conditions in remote Australian Indigenous communities. As in developing countries, this approach has largely failed. There are a number of proposed explanations for this failure. One hypothesis is that any improvement in the physical system for water supply will not necessarily translate into health benefits as half measures can aggravate conditions and low levels of investments can actually increase the risks to health [75]. In the case of remote Australian Indigenous communities, the provision of additional and improved housing has been the major focus for improving the health of Indigenous people for the past 40 years. The newly constructed houses mostly consisted of three bedrooms, one bathroom and toilet, plus kitchen and living area. In the rush to construct new dwellings issues surrounding maintenance of health hardware and the need to adapt living practices for this new environment received only hasty consideration, with few having insight into the potential problems that might arise [76]. The rapid introduction of new technology, crowding, and problems caused by a failure to change behaviours to suit the new housing environment, lead to another layer of complexity when trying to promote hygiene. Tatz's [18] (p. 113) observations of what occurred in the 1960s was that either no education or "un-empathetic or accommodating" education was provided.

It has also been suggested that the 'threshold-saturation theory' may help to explain the relationship between water supply, sanitation investments and health and conflicting empirical findings [77]. That is, that the relationship between access to water and sanitation interventions, and improved health among low socio-economic groups in developing countries, is not linear but the relationship is better explained as an S-shaped logistic curve. This way the important biological and social phenomena that have both threshold and saturation level characteristics can be better taken into account. Shuval et al [77] (p. 246) describe an initial lag or threshold phase for communities at the lowest socio-economic levels. They believe that improvements in water and sanitation infrastructure in these communities are unlikely to cause any improvement in health status because of multiple routes of disease transmission, poor nutrition, improper personal hygiene, low resistance to disease and heavily contaminated living environments. They propose that for communities at the lower end of the socio-economic scale, in the range below the threshold, that an effective health promotion policy would need to provide, in addition to infrastructure, an "integrated, broad-spectrum programme" that includes sanitation, nutrition, education and primary health care, coupled with efforts to encourage general economic and social development. More recently Roberts [78] restated this argument. Given that housing and water and sanitation technology are considered to have been introduced into remote Indigenous communities in an ad hoc manner [18], this hypothesis may explain why children's health in these communities has been slow to improve. While the socio-economic situation of Indigenous Australians is not directly comparable with that in developing country populations, using different social determinants of health, for example taking into account historical events, rapid acculturation and high levels of family and community dysfunction, the level of extreme disadvantage might be considered comparable and the threshold-saturation theory may apply.

An alternative hypothesis is that high exposure to infection and poor domestic hygiene is keeping Indigenous children at threshold level, while some small improvement will commence them moving up the curve. It is difficult to determine where remote Indigenous communities lie in relation to the threshold-saturation theory. However, if it is accepted that currently most communities are well below the threshold then it follows (according to the 'threshold-saturation theory') that major investments are needed to have an impact on health outcomes. However, unlike developing countries, most remote Indigenous communities have relatively functional water and sanitation systems. Therefore, what is likely to be most needed in many remote communities are not investments in new major water and sanitation systems, but rather a co-ordinated approach to ensure the current systems and household technologies are well maintained and are used appropriately (or investments in improvements in hygiene rather than water and sanitation).

In case of the need to choose one intervention that is likely to have most benefit, this review has shown that there is good evidence to support that handwashing with soap after defecating and before eating is effective in reducing rates of diarrhoea. Therefore, this intervention should be included in all hygiene improvement programs whether intended for Indigenous or non-Indigenous communities. However, in remote communities, where high rates of disease among children reflect serious environmental contamination, the need for handwashing with soap is much greater. In addition to reducing rates of diarrhoeal disease, handwashing with soap offers some additional benefits. Luby et al [79] subsequently published additional study results that show that their intervention (education and handwashing with soap, and encouraging frequent bathing) resulted in a 34% lower incidence of impetigo (95% CI 0.48, 0.84), and a 50% lower incidence of pneumonia (95% CI 0.35, 0.66) among children <5 years compared to the control group. However, while this intervention is effective under study conditions, replicating the methodology in remote Indigenous communities would be challenging given its cost. It is unlikely that the same good effect could be achieved in the same timeframe (12 months). Luby et al [50] acknowledge that the cost of delivering their intervention was very high and that it is not feasible to replicate in the practice setting. In this study every household was visited at least weekly by fieldworkers who provided them with an unlimited supply of soap at no cost and motivated them to change their behaviour. While the cost of implementing this intervention is likely to be barrier to its use across a number of remote Indigenous communities, other cultural, social and political factors would come into play. Raising sensitive issues such as the need for hygiene improvement can be perceived as confrontational by individuals or communities and can be seen as 'victim-blaming'. Until more recently, the tensions surrounding issues such as poor hygiene and unsanitary living conditions have caused authorities to allow unacceptable public health risks continuing in remote Indigenous communities without taking effective action.

Evidence of effectiveness is an important criterion with which to identify and choose what hygiene interventions to use. However, other factors also need to be considered, for example, many interventions shown to be efficacious in one community setting have not achieved the same effect in a different community setting [80-82]. Community-specific factors that may need to be considered include current hygiene practices, ethical implications, geography, cost, feasibility, the likely acceptability of the intervention to the community, local and broader political contexts, and taking account of the advice of those already experienced in the field. The problems that underlie unsanitary living conditions and poor hygiene in remote communities make it unlikely that a single intervention is sufficient to reduce the rate of infections experienced by children. A theory of multifactorial etiology, including biological as well as socio-economic and psychosocial factors, is now considered essential to establish sound and effective public health policies [83]. "Ecological" interventions present significant challenges to evaluate, including difficulties in demonstrating direct association between interventions and health outcomes at the individual level. Nevertheless, our assessment is that the effects of multifaceted interventions, for example improved housing, reduced household crowding and hygiene promotion are likely to provide the greatest opportunity to improve hygiene in remote Indigenous communities [84].

## Conclusion

The findings of this systematic review indicate that research aimed at measuring the effectiveness of hygiene interventions is complex and challenging. There was a need to draw heavily on research conducted in developing countries as little or no research in the Indigenous Australian context was available. The high burden of infection experienced by children living in remote Indigenous communities indicates that more research urgently needs to be undertaken in this area. The studies conducted overseas describe substantial benefits associated with interventions that target handwashing with soap.

## Competing interests

The authors declare that they have no competing interests.

## Authors' contributions

EM developed the study design, was a reviewer and wrote the draft manuscript. RB was a reviewer and had major role in revising the manuscript. DB was a reviewer and assisted in revising the manuscript. PM was a reviewer and assisted in revising the manuscript. All authors read and approved the final manuscript.

## Pre-publication history

The pre-publication history for this paper can be accessed here:


